# Inhibition of p53 deSUMOylation Exacerbates Puromycin Aminonucleoside-Induced Apoptosis in Podocytes

**DOI:** 10.3390/ijms151121314

**Published:** 2014-11-18

**Authors:** Lingyu Wang, Jingwei Zhu, Ming Fang, Tuaner Zhang, Hua Xie, Nan Wang, Nan Shen, Hui Guo, Bo Fu, Hongli Lin

**Affiliations:** 1 Department of Nephrology, Liaoning Translational Medicine Center of Nephrology, the First Affiliated Hospital of Dalian Medical University, Dalian 116011, China; E-Mails: wanglingyu0622@163.com (L.W.); zjwssss2011@163.com (J.Z.); fangming0411@126.com (M.F.); dlzhangtuaner@163.com (T.Z.); wlciq007@163.com (H.X.); pyran78@163.com (N.W.); sn106@hotmail.com (N.S.); dahuihui800@163.com (H.G.); 2 Department of Nephrology, State Key Laboratory of Kidney Disease, PLA General Hospital, Beijing 100853, China; E-Mail: fubo_01@163.com

**Keywords:** apoptosis, deSUMOylation, p53, podocyte, sentrin/SUMO-specific protease 1

## Abstract

Apoptosis is a major cause of reduced podocyte numbers, which leads to proteinuria and/or glomerulosclerosis. Emerging evidence has indicated that deSUMOylation, a dynamic post-translational modification that reverses SUMOylation, is involved in the apoptosis of Burkitt’s lymphoma cells and cardiomyocytes; however, the impact of deSUMOylation on podocyte apoptosis remains unexplored. The p53 protein plays a major role in the pathogenesis of podocyte apoptosis, and p53 can be SUMOylated. Therefore, in the present study, we evaluated the effect of p53 deSUMOylation, which is regulated by sentrin/SUMO-specific protease 1 (SENP1), on podocyte apoptosis. Our results showed that SENP1 deficiency significantly increases puromycin aminonucleoside (PAN)-induced podocyte apoptosis. Moreover, SENP1 knockdown results in the accumulation of SUMOylated p53 protein and the increased expression of the p53 target pro-apoptotic genes, *BAX*, *Noxa* and *PUMA*, in podocytes during PAN stimulation. Thus, SENP1 may be essential for preventing podocyte apoptosis, at least partly through regulating the functions of p53 protein via deSUMOylation. The regulation of deSUMOylation may provide a novel strategy for the treatment of glomerular disorders that involve podocyte apoptosis.

## 1. Introduction

Clinical and experimental studies have revealed that a decrease in podocyte number is closely associated with the initiation of glomerulosclerosis and contributes to the progression of chronic kidney disease (CKD) [1,2,3,4]. Apoptosis is a major cause of reduced podocyte numbers in glomerular diseases [5,6,7]. At present, there is no satisfactory method or target for inhibiting podocyte apoptosis. Thus, the investigation of the pathogenesis of podocyte apoptosis is important to develop novel therapeutic strategies for CKD. 

SUMOylation is a crucial post-translational modification of targeted proteins, which is characterized by the covalent conjugation of a small peptide (SUMO) [8,9]. DeSUMOylation is the reverse process in which substrate proteins deconjugate the SUMO peptide via sentrin/SUMO-specific proteases (SENPs) [10]. SUMOylation/deSUMOylation is a highly dynamic process that regulates various physiological processes, including cell growth, differentiation, senescence, oxidative stress and apoptosis [11]. Over recent years, the effect of deSUMOylation has been demonstrated in carcinogenesis and tumor progression [12,13,14]. However, studies on the role of deSUMOylation in kidney disease are nonexistent. 

p53 has been identified as a pivotal factor that regulates the apoptosis of podocytes [15]. The p53 protein is inactive in normal cells unless cells are exposed to various stress signals, which promotes the activation of p53 protein by post-translational modifications [16,17,18]. Interestingly, several studies have shown that p53 could be modified by SUMO-1, and the consequent SUMOylation induces the pro-apoptotic activity of p53 and downstream pro-apoptotic gene transcription [19,20,21,22]. However, the mechanism of p53 deSUMOylation remains unclear. Accordingly, we chose the typical p53 pathway to explore the effect of deSUMOylation on podocyte apoptosis by inhibiting SENP1, the most effective deSUMOylate protease [23].

In this study, we established an *in vitro* model of apoptotic podocytes using puromycin aminonucleoside (PAN), which is widely used to induce podocyte apoptosis both *in vivo* and *in vitro*. We observed that SENP1 deficiency resulted in a significant increase in the rate of podocyte apoptosis induced by PAN. The inhibition of SENP1 also promoted the accumulation of SUMOylated p53 protein and the transcription of p53-dependent pro-apoptotic genes, including *BAX*, *Noxa* and *PUMA*, that are otherwise induced by PAN stress. Our results indicate that SENP1 may contribute to preventing podocytes from undergoing PAN-induced apoptosis. This provides new insights into the mechanism of glomerular dysfunction, and suggests that the deSUMOylation of the p53 protein might represent a novel therapeutic strategy for the treatment of glomerular disorders.

## 2. Results

### 2.1. SENP1 and p53 Expression Increased and Accompanied Podocyte Apoptosis Induced by PAN

**Figure 1:**
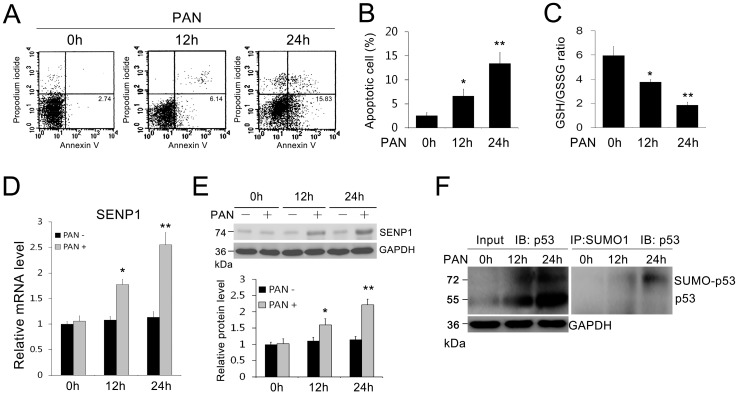
SENP1 expression increased in podocytes after PAN treatment. (**A**) Representative flow cytometric analysis of apoptotic cells stained with Annexin V-FITC and propidium iodide (PI) after treatment with PAN (5 μg/mL) for 0, 12 and 24 h. Annexin V-positive cells were defined as apoptotic cells; (**B**) The apoptosis rates of MPC5 cells in each group. All data are presented as the mean ± SEM of three independent experiments. * *p* < 0.05 and ** *p* < 0.01 *vs.* 0 h group; (**C**) The GSH/GSSG ratio was analyzed in cultured MPC5 cells. The GSH/GSSG ratio was down-regulated by PAN treatment. * *p* < 0.05 and ** *p* < 0.01 *vs.* 0 h group; (**D**) Real-time PCR of SENP1 mRNA in MPC5 cells at indicated times after PAN treatment (5 μg/mL). All data are presented as the mean ± SEM of three independent experiments. * *p* < 0.05, ** *p* < 0.01 *vs.* 0 h group; (**E**) Western blotting of SENP1 protein expression in MPC5 cells at the indicated times after PAN treatment (5 μg/mL). All data are presented as the mean ± SEM of three independent experiments. * *p* < 0.05, ** *p* < 0.01 *vs.* PAN(–) group; (**F**) The SUMOylated form of p53 protein was determined by immunoprecipitation with an anti-SUMO1 antibody followed by Western blotting with an anti-p53 antibody. GAPDH was used as the control. This experiment was performed in triplicate.

To test the presence of PAN-induced apoptosis in podocytes, we initially detected the amount of apoptosis in MPC5 cells using FACS analysis and found that apoptotic MPC5 cells progressively increased after PAN treatment (Figure 1A,B). We also found that the Glutathione/Oxidized Glutathione (GSH/GSSG) ratio, which reflects the cellular oxidative stress, was progressively down-regulated by PAN treatment (Figure 1C). As it is unclear whether PAN stimulation leads to changes in SENP1 expression in podocytes, we simultaneously examined the expression of SENP1 in PAN-treated MPC5 cells. We observed low levels of SENP1 in normal podocytes; however, after cells were incubated with PAN for 12 and 24 h, the levels of SENP1 mRNA (Figure 1D) and protein (Figure 1E) in MPC5 cells were upregulated along with increased levels of podocyte apoptosis. Furthermore, we examined changes in the levels of SUMOylated p53 in PAN-treated MPC5 cells and found that SUMOylated and de-SUMOylated p53 were all increased progressively after PAN treatment (Figure 1F). These results suggested that SUMOylation/deSUMOylation of p53 protein associated with podocyte apoptosis induced by PAN. SENP1 might be involved in this process by regulating SUMOylation/deSUMOylation of p53.

### 2.2. Establishment of SENP1-Knockdown in MPC5 Cells

**Figure 2:**
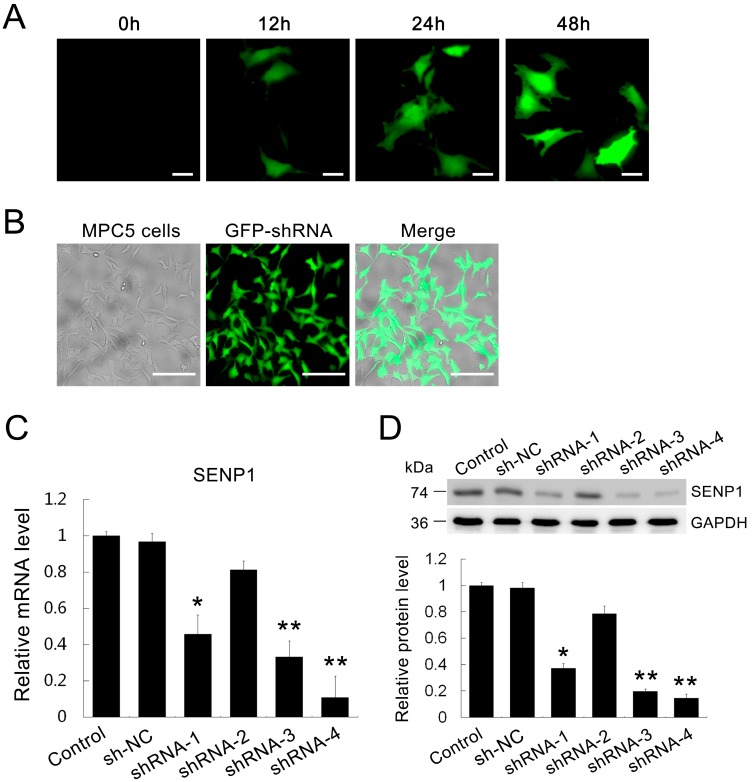
Knockdown effect of SENP1 shRNA plasmids transfection in MPC5 cells. MPC5 cells were transfected with scrambled shRNA and SENP1 shRNA plasmids. (**A**) Representative photomicrographs show GFP-labeled scrambled shRNA plasmid expression and localization in MPC5 cells before (0 h) and at 12, 24, and 48 h after transfection. Bar = 25 μm; (**B**) Representative photomicrographs show that the transfection efficiency was more than 80% at 48 h. Bar = 100 μm; (**C**) Real-time RT-PCR and (**D**) Western blotting indicate that SENP1 shRNAs inhibited the expression of both mRNA and protein of endogenous SENP1. All data are presented as the mean ± SEM of three independent experiments. * *p* < 0.05, ** *p* < 0.01 *vs.* control group.

To address the role of SENP1 in the PAN-induced apoptosis of podocytes, we sought to downregulate SENP1 using RNA interference in MPC5 cells. Scrambled short hairpin RNA (shRNA) plasmid as the negative control (NC) and four SENP1 shRNA plasmids that targeted the SENP1 gene were independently transfected into MPC5 cells. The expression of exogenous GFP-labeled shRNA in MPC5 cells was detectable at 12 h and reached its peak value at 48 h after transfection (Figure 2A). The results of real-time PCR and western blotting confirmed that SENP1 shRNA-4 was the most efficient shRNA to decrease SENP1 mRNA (Figure 2B) and protein levels (Figure 2C,D) in MPC5 cells.

### 2.3. SENP1 Knockdown Increased PAN-Induced Apoptosis in Podocytes

**Figure 3:**
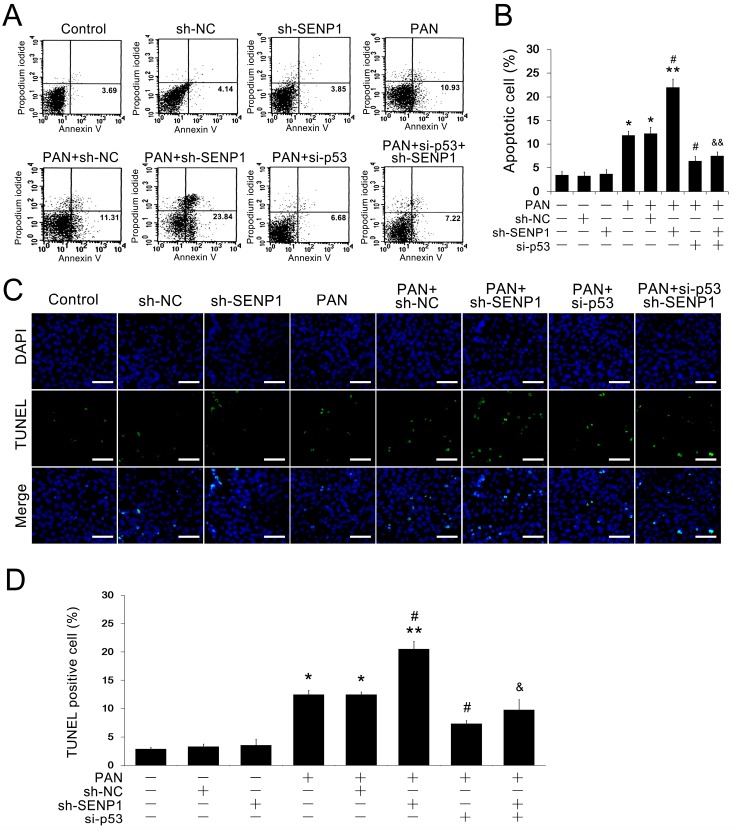
Knockdown of SENP1 promotes PAN-induced apoptosis in MPC5 cells. (**A**) Representative flow cytometric analysis of apoptotic cells stained with Annexin V-FITC and PI after treatment with 5 μg/mL PAN for 24 h. Annexin V-positive cells were defined as apoptotic cells; (**B**) The apoptosis rates of MPC5 cells in each group. All data are presented as the mean ± SEM of three independent experiments. * *p* < 0.05 and ** *p* < 0.01 *vs.* control group; ^#^ *p* < 0.05 *vs.* PAN or PAN + sh-NC groups; ^&&^ *p* < 0.01 *vs.* PAN + sh-SENP1 group; (**C**) Representative images of TUNEL/DAPI staining are shown to illustrate apoptotic podocytes. Bar = 100 μm. All results are presented as the mean ± SEM of three independent experiments; (**D**) The rates of TUNEL positive MPC5 cells in each group. All data are presented as the mean ± SEM of three independent experiments. * *p* < 0.05 and ** *p* < 0.01 *vs.* control group; ^#^ *p* < 0.05 *vs.* PAN or PAN + sh-NC groups; ^&^ *p* < 0.05 *vs.* PAN + sh-SENP1 group.

To determine the role of SENP1 in influencing the apoptosis of podocytes, we initially quantified PAN-induced apoptosis in MPC5 cells with or without the transfection of the selected SENP1 shRNA by FACS analysis. As shown in Figure 3A,B, the rate of apoptosis of normal MPC5 cells increased to 11.83% ± 0.89% 24 h after PAN treatment (5 μg/mL). This effect was enhanced in SENP1 knockdown MPC5 cells, in which the rate of apoptosis was significantly increased by up to 1.86-fold (from 22.02% ± 1.73%). Then we used p53 siRNA to blockade the p53 pathway to directly examine whether SENP1 knockdown induced apoptosis was regulated by p53. After the p53 knockdown, SENP1 shRNA failed to induce apoptosis in podocytes during PAN treatment (Figure 3A,B). We also detected apoptosis by TUNEL/DAPI double staining, which showed an increased number of apoptotic cells in addition to enhanced nuclear staining and apoptotic bodies in SENP1 knockdown MPC5 cells compared to wild-type PAN-treated MPC5 cells (Figure 3C,D). Furthermore, this effect was markedly reversed by p53 inhibition in SENP1 knockdown MPC5 cells, in which the TUNEL positive podocytes decreased (Figure 3C,D). These results indicated that SENP1 may be essential for podocyte survival during PAN-induced stress and the effect of SENP1 on regulating podocytic apoptosis may be mediated by the p53 pathway.

### 2.4. SENP1 Deficiency Induces Apoptosis in Podocytes through p53 SUMOylation

**Figure 4:**
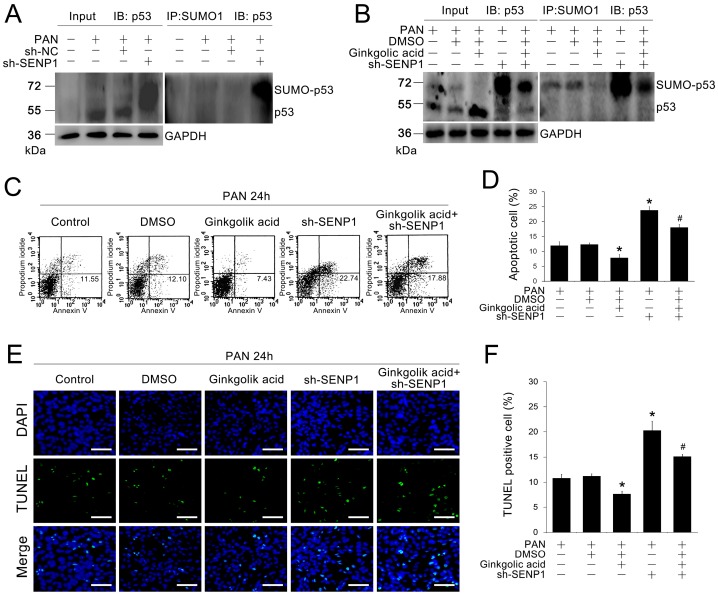
SENP1 knockdown induces apoptosis in podocytes through p53 SUMOylation. (**A**,**B**) Immunoprecipitation analysis was performed to detect SUMOylation and deSUMOylation levels of p53 protein in MPC5 cells. SUMOylated p53 was increased in the SENP1 shRNA group after treatment with 5 μg/mL PAN for 24 h. MPC5 cells transfected with SENP1 shRNA plasmid were treated with DMSO only for control or with 2 μM ginkgolic acid. This experiment was performed in triplicate; (**C**) Representative flow cytometric analysis of apoptotic cells stained with Annexin V-FITC and PI after treatment with 2 μM ginkgolic acid in SENP1 knockdown MPC5 cells. Annexin V-positive cells were defined as apoptotic cells; (**D**) The apoptosis rates of MPC5 cells in each group. All data are presented as the mean ± SEM of three independent experiments. * *p* < 0.05 *vs.* PAN or PAN + sh-NC groups; ^#^ *p* < 0.05 *vs.* PAN + sh-SENP1 group; (**E**) Representative images of TUNEL/DAPI staining are shown to illustrate apoptotic podocytes. Bar = 100 μm. All results are presented as the mean ± SEM of three independent experiments; (**F**) Rates of TUNEL positive MPC5 cells in each group. All data are presented as the mean ± SEM of three independent experiments. * *p* < 0.05 *vs.* control group; ^#^ *p* < 0.05 *vs.* PAN or PAN + sh-NC groups.

To determine whether the effect of SENP1 on podocyte apoptosis is related to the p53 pathway, we examined changes in the levels of SUMOylated and de-SUMOylated p53 protein in SENP1 knockdown MPC5 cells. As shown in Figure 4A, we observed that the SUMOylation of p53 protein increased significantly in SENP1 knockdown MPC5 cells compared with normal MPC5 cells after PAN stimulation, which suggested that p53 could be deSUMOylate-modified in a process that was directly regulated by SENP1 in podocytes. Next, we treated SENP1 knockdown MPC5 cells with ginkgolic acid, a known SUMOylation inhibitor [24]. After ginkgolic acid treatment, SUMOylated p53 level was decreased in SENP1 knockdown MPC5 cells (Figure 4B). Moreover, the apoptosis rate of SENP1 knockdown MPC5 cells was also reduced after ginkgolic acid treatment (Figure 4C–F), which suggested that SENP1 deficiency induced apoptosis in podocytes may be directly mediated through p53 SUMOylation.

### 2.5. SENP1 Deficiency Induces the Upregulation of p53-Dependent Pro-Apoptotic Genes in Podocytes

**Figure 5:**
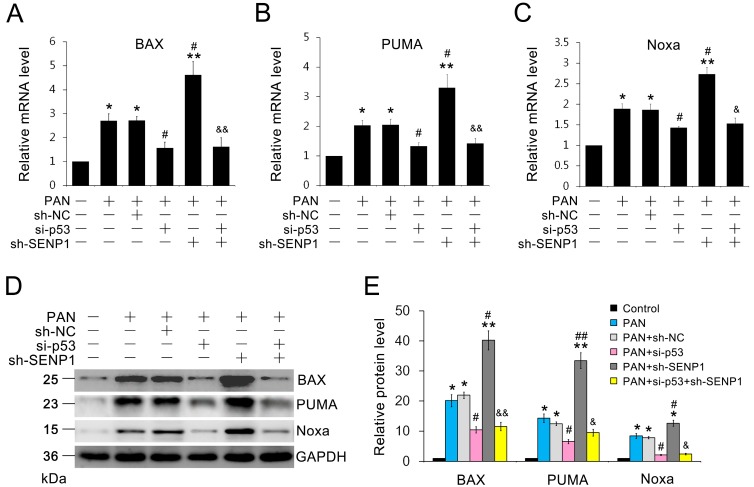
Blockade of SENP1 results in upregulation of p53-dependent pro-apoptotic genes in podocytes. (**A**–**C**) Real-time RT-PCR indicates that the knockdown of SENP1 induced the expression of *BAX*, *Noxa* and *PUMA* mRNA in PAN-treated MPC5 cells. All data are presented as the mean ± SEM of three independent experiments. * *p* < 0.05, ** *p* < 0.01 *vs.* control group; ^#^ *p* < 0.05 *vs.* PAN or PAN + sh-NC groups; ^&^ *p* < 0.05, ^&&^ *p* < 0.01 *vs.* PAN + sh-SENP1 group; (**D**,**E**) Western blotting analysis of *BAX*, *Noxa* and *PUMA* protein levels in PAN-treated MPC5 cells. All data are presented as the mean ± SEM of three independent experiments. * *p* < 0.05, ** *p* < 0.01 *vs.* control group; ^#^ *p* < 0.05, ^##^ *p* < 0.01 *vs.* PAN or PAN + sh-NC groups; ^&^ *p* < 0.05, ^&&^ *p* < 0.01 *vs.* PAN + sh-SENP1 group.

We measured the mRNA and protein levels of pro-apoptotic p53 target genes, including *BAX*, *Noxa* and *PUMA*, which were all identified as effectors of p53-mediated apoptosis [25,26,27,28,29]. After PAN stimulation, the mRNA and protein expression of *BAX*, *Noxa* and *PUMA* were significantly increased in SENP1-deficient MPC5 cells compared to wild-type MPC5 cells (Figure 5A–E). To directly demonstrate whether SENP1 knockdown induced pro-apoptotic gene expression was p53 dependent, we used p53 siRNA to blockade the p53 pathway. After the p53 knockdown, SENP1 shRNA failed to induce up-regulation of *BAX*, *Noxa* and *PUMA* expression during PAN treatment (Figure 5A–E), which indicated the effect of SENP1 on regulating pro-apoptotic gene expression through the p53 pathway in podocytes. All of the above data indicated that SENP1 plays a critical role in preventing the pro-apoptotic activity of p53 in podocytes.

## 3. Discussion

This study has provided novel insights into the pathogenesis of podocyte apoptosis by probing the role of the post-translational modifications, SUMOylation and deSUMOylation, in kidney disease for the first time. We found that SENP1 was expressed at low levels in normal podocytes, and was markedly increased in podocytes subjected to PAN-induced apoptosis and oxidative stress (Figure 1). Moreover, we also found that SUMO-1 conjugated p53 (SUMOylated p53), the pivotal factor that regulates podocyte apoptosis, was accumulated after PAN treatment. Accordingly, we hypothesized that SENP1 might be involved in PAN-induced podocyte apoptosis, which is probably associated with the p53 pathway. Previous studies have indicated that SENP2 plays a role in p53 deSUMOylation and p53-mediated cellular apoptosis [30,31]. As SENP1 is shown to have a greater efficiency to deconjugate SUMO-1 than other family members [23,32], it is conceivable that SENP1 also has a crucial role in p53-induced apoptosis, which remains to be explored.

To further elucidate the role of SENP1 in podocyte apoptosis, we designed and synthesized a SENP1 shRNA plasmid and transfected it into MPC5 cells. Then, we detected the degree of apoptosis in normal podocytes and SENP1 knockdown podocytes after PAN stimulation to determine whether SENP1 deficiency actually results in the increased apoptosis of podocytes. After PAN treatment for 24 h, the rate of apoptosis in normal podocytes only increased from 3.47% ± 0.71% to 11.83% ± 0.89%, while the rate of apoptosis in SENP1 knockdown podocytes increased by up to 22.02% ± 1.73%, almost 2-fold more than normal podocytes (Figure 3A,B). TUNEL staining also showed a significant increase in the number of apoptotic cells in SENP1 knockdown podocytes compared to normal podocytes under PAN-induced stress (Figure 3C,D). Importantly, we also found that the effect of SENP1 on regulating apoptosis in podocytes was p53-dependent (Figure 3), indicating the essential role of p53 in SENP1 deficiency induced podocytic apoptosis during PAN stress. Our data demonstrated that the knockdown of SENP1 rendered podocytes more susceptible to PAN-induced injury and enhanced the degree of PAN-induced podocyte apoptosis. Therefore, the upregulation of SENP1 during PAN stimulation might be a compensatory reaction in podocytes for deSUMOylating p53 protein. The growth in the number of apoptotic podocytes may be explained if the upregulation of SENP1 is still insufficient for deSUMOylation of all SUMOylated p53 protein. These findings suggested that SENP1 is essential for podocyte survival during PAN-induced cellular stress.

Jiang *et al.* [33] previously reported that SENP1 had a critical contribution in protecting MEF cells from endoplasmic reticulum (ER) stress-induced apoptosis via X box-binding protein 1 (XPB1). Huang *et al.* [34] showed that SENP1 inhibition promoted cell apoptosis in Burkitt’s lymphoma cells. Although these studies suggest the role of SENP1 in preventing cellular apoptosis, our study is the first to focus on the role of SENP1 in podocyte apoptosis. Furthermore, Yates *et al.* have reported that inhibition of SENP1 induced p53-dependent premature senescence in human fibroblasts [35]. However, few studies have investigated the relationship between SENP1 and typical p53-dependent apoptosis.

Accordingly, we next determined whether SENP1 is responsible for regulating p53 deSUMOylation in podocytes. As shown in Figure 4A, the SUMOylation of p53 was significantly increased in SENP1 knockdown podocytes compared with normal podocytes after PAN treatment. Furthermore, after treatment with ginkgolic acid, a known SUMOylation inhibitor, the SUMOylated form of p53 protein was decreased accompany with diminished apoptotic rate in SENP1 knockdown podocytes. Above results suggested that a deficiency of SENP1 could results in aggravating apoptosis in PAN-treated podocytes, which was directly mediated by accumulation of SUMOylated p53.

It has been reported that p53 promotes cellular apoptosis via the direct activation of its downstream pro-apoptotic genes [36,37]. To prove that the possible protective effect of SENP1 against apoptosis is specifically mediated through p53 signaling, we measured the level of p53-dependent pro-apoptotic gene expression in SENP1 knockdown podocytes. *BAX*, *Noxa* and *PUMA*, which have all been identified as p53 target genes that are upregulated during cellular apoptosis, have been shown to be essential mediators of p53-dependent apoptotic pathways [25,26,27,28,29]. Therefore, we used real-time PCR and western blotting to determine the transcription and protein levels of these three genes. The data showed that their expression in both mRNA (Figure 5A–C) and protein levels (Figure 5D,E) in SENP1 knockdown podocytes were significantly higher than in normal podocytes after PAN treatment. Furthermore, after p53 knockdown, SENP1 deficiency failed to upregulate the expression of the three pro-apoptotic factors, indicating the essential role of p53 in mediating the SENP1 deficiency induced podocyte apoptosis. Several studies have reported that SUMOylation by SUMO-1 could promote the transcriptional activity of p53 during cellular stress [38,39]. All of the above results indicate that SENP1 protects podocytes from PAN-induced apoptosis via the deSUMOylation of p53. Therefore, we hypothesize that repressing p53 signaling could be useful for the treatment of glomerular disorders by targeting its effects on SENP1.

In recent years, the post-translational SUMOylation and deSUMOylation of proteins have become important and attractive biomarkers and treatment targets in various cancers [12,13,14]. The increased expression of SUMO proteases is found in various tumor cell types and has been correlated with various aspects of carcinogenesis and tumor progression [40,41,42]. In addition, several studies have indicated that SENP1-mediated deSUMOylation is protective against oxidative stress in cardiomyocytes and neuroblastoma cells [43,44]. However, the potential effects of SUMOylation and deSUMOylation on kidney diseases have not been explored. The glomerular visceral epithelial cell (podocyte) is regarded as a terminally differentiated and highly specialized cell with limited capacity for cell replication. The decrease in podocyte number is closely associated with the initiation of glomerulosclerosis and contributes to the progression of chronic kidney disease. Apoptosis is a major cause of reduced podocyte numbers in glomerular diseases. But so far, there is no satisfactory method or target for inhibiting podocyte apoptosis. Our present study provides a new insight into the pathogenesis of podocyte apoptosis, which could be a potential therapeutic target for kidney diseases.

According to recent studies, SENP1 deficiency also results in promotion of cellular apoptosis via increased SUMOylation of other molecular targets, including X box-binding protein 1 (XBP1) [33] and hypoxia inducible factor 1 (HIF-1) [34]. Therefore, we cannot exclude the possibility that other targets of SENP1 may be involved in this pro-apoptotic effect in our experiments. Further studies should be performed to better define the regulation mechanisms of SENP1 in apoptotic signaling crosstalk.

Although our present study revealed the beneficial effects of SENP1 mediated deSUMOylation on anti-apoptosis in podocytes, there are still some different standpoints in other studies which could not be ignored. Meinecke *et al.* reported that increased levels of SUMOylation of nuclear promyelocytic leukemia (PML) protein in rheumatoid arthritis synovial fibroblasts contributed to the resistance of these cells against Fas-induced apoptosis [45]. Li *et al.* showed that SENP1 mediated HIPK1 deSUMOylation in TNF-treated human umbilical vein endothelial cells (HUVECs), which leads to enhanced ASK1-dependent apoptosis [46]. These conflicting results are very interesting and may indicate the different cell types studied, in addition to varying types of stimulation and studied signaling pathways. Further studies should therefore be performed to explore the relationship between SUMOylation/deSUMOylation and cellular apoptosis.

In addition, other post-transcriptional modifications of p53 also influence p53 activity and associated with cellular apoptosis, such as phosphorylation, methylation, acetylation and ubiquitination [17,18]. It has been shown that phosphorylation of mouse p53 at Ser18, Ser23 and Ser46 are critical to activate p53-dependent apoptosis after DNA damage [47,48]. Moreover, monoubiquitination is also shown to have great potential to regulate p53 conformation and mitochondrial translocation, which promotes the mitochondrial apoptotic program of p53 [49,50]. Accordingly, the underlying mechanism in regulation of p53 activity and pro-apoptotic effects seems to be a complex network, requiring further investigation and elucidation.

In summary, our study is the first to reveal the beneficial effects of SENP1 on the treatment of kidney diseases. We show the specific role of SENP1 in regulating p53 deSUMOylation and p53-dependent apoptotic genes transcription during PAN-induced podocyte injury. This study suggests that the post-translational modification of key molecules by SUMOylation and deSUMOylation could provide a novel strategy for the treatment of podocyte disorders in glomerular diseases.

## 4. Experimental Section

### 4.1. Cell Culture

Immortalized mouse podocytes (MPC5) were kindly provided by Professor Peter Mundel of the Medical College of Harvard University (Boston, MA, USA). Undifferentiated MPC5 cells were cultured in RPMI-1640 medium supplemented with 10% fetal bovine serum (Hyclone), 100 U/mL penicillin, 100 μg/mL streptomycin and 20 U/mL recombinant mouse interferon-γ (PeproTech, Rocky Hill, NJ, USA) at 33 °C for proliferation. Cells were differentiated at 37 °C under interferon-γ depletion for 10–14 days [51]. The cells were cultured synchronously in medium containing 0.2% FCS and 5.5 mM d-glucose RPMI-1640 medium for 24 h before the experiment. To induce MPC5 cell apoptosis, PAN was added to the medium at a concentration of 5 μg/mL. Ginkgolic acid (Calbiochem, Darmstadt, Germany) was dissolved in DMSO and added to the medium at a concentration of 2 μM.

### 4.2. Small Hairpin RNA (shRNA) Plasmids, Small Interfering RNA(siRNA) and Transient Transfections

Four shRNAs targeting the *SENP1* gene, which were identified using BLAST and the mouse genome database to assess possible cross-reactivity, were synthesized by Genepharma (Shanghai, China). The cDNAs encoding the four mouse SENP1 shRNAs (SENP1-1, 5'-GCAGGATCCTCTTGCAATACC-3'; SENP1-2, 5'-GCACCTCATCAGCCAAATAGC-3'; SENP1-3, 5'-GCATTCCGCTTGACCATTACA-3'; SENP1-4, 5'-GCAGTGAAACG CTGGACAAAG-3') were inserted independently into the recombinant plasmid pGPU6. The p53 siRNA was designed using the BLOCK-iT RNAi Designer software and has been proved to be effective in *p53* gene silencing. The sequences of the p53 siRNA are 5'-CACCUCACUGCAUGGACGAUCUGUU-3' for sense and 5'-AACAGAUCGUCCAUGCAGUGAGGUG-3' for antisense. For *SENP1* and *p53* gene silencing, cells were transfected with SENP1 shRNA plasmid or p53 siRNA using Lipofectamine 2000 (Invitrogen, Carlsbad, CA, USA). A scrambled shRNA plasmid was used as the mock shRNA.

### 4.3. Protein Isolation and Immunoblotting

Cells from different groups were collected, dissolved in RIPA buffer and centrifuged at 15,000× *g* for 10 min at 4 °C. The supernatants were collected, and the cellular protein concentrations were determined with a BCA protein assay kit (Pierce Biotechnology, Rockford, IL, USA). Protein samples were denatured at 95 °C for 5 min, separated by SDS-PAGE and electrophoretically transferred to polyvinylidene difluoride membranes (Millipore, Billerica, MA, USA). The blots were incubated with primary antibodies against SENP1 (1:1000; Abcam, Cambridge, UK) and p53 (1:200; Santa Cruz Biotechnology, Santa Cruz, CA, USA) overnight at 4 °C. Subsequently, the blots were incubated with a horseradish peroxidase labeled secondary antibody (1:5000; Zhongshan Biotechnology, Beijing, China) for 2 h at 37 °C. The bands were visualized using an ECL kit (Amersham-Pharmacia Biotech, Little Chalfont, UK) and quantified with Labworks™ Image Analysis software (UVP, Upland, CA, USA).

### 4.4. Immunoprecipitation

Cell pellets were washed three times with cold 0.01 M PBS and lysed in Radioimmune Precipitation Assay (RIPA) buffer (Laiwen, Jiangsu, China) for 30 min on ice. After centrifugation at 12,000 rpm for 15 min at 4 °C, the lysates were collected and precleared by Protein G PLUS-Agarose (Santa Cruz Biotechnology, Santa Cruz, CA, USA) and then incubated with an anti-SUMO1 antibody (1:50; Cell Signaling Technology, Boston, MA, USA) with continual shaking for 2 h. The protein-antibody complexes were collected with 20 μL of Protein G PLUS Agarose at 4 °C with continual shaking overnight. Subsequently, the immunoprecipitates were washed three times with lysis buffer and analyzed by 12% SDS-PAGE and immunoblotting.

### 4.5. RNA Isolation and Real-Time RT-PCR

Total RNA was extracted from each sample, and the first-strand cDNA synthesis was performed using a SYBR^®^ PrimerScript™ RT-PCR Kit (Takara, Otsu, Shiga, Japan). Real-time PCR was performed to determine relative mRNA levels using primers described in Table 1 on the ABI 7500 fast system (Applied Biosystems, Foster City, CA, USA). PCR cycling conditions included an initial step at 95 °C for 30 s, followed by 40 cycles of 5 s at 95 °C, 20 s at 60 °C and 30 s at 72 °C. The PCR products were assessed by melting curve analysis, and gene expression levels were calculated using the ^ΔΔ^*C*_t_ method after normalization to the *GAPDH* housekeeping gene. All PCR samples were tested in triplicate.

**Table 1:** Real-time RT–PCR primer sequences.

Gene/Primer	Direction	Sequence (5' to 3')
*SENP1*	Forward	CTACAAGAAGCCCAGCCTATCGTC
	Reverse	GTCACCTGAGCCAAGGAAACTG
*BAX*	Forward	ACCAAGAAGCTGAGCGAGTGT
	Reverse	ACAAACATGGTCACGGTCTGC
*Noxa*	Forward	GCAGAGCTGGAAGTCGAGTGT
	Reverse	AAGTTTCTGCCGGAAGTTCAG
*PUMA*	Forward	ACGACCTCAACGCACAGTACG
	Reverse	TCCCATGATGAGATTGTACAGGAC
*GAPDH*	Forward	GCACCGTCAAGGCTGAGAAC
	Reverse	TGGTGAAGACGCCAGTGGA

### 4.6. FACS Analysis

Cells from different groups were harvested, washed with PBS and resuspended in 500 μL binding buffer. The Annexin V-FITC/PI Apoptosis Detection Kit (KeyGEN BioTECH, Nanjing, China) was used according to the manufacturerʼs protocol. The analysis of cell apoptosis was performed using the FACSVantage SE Flow Cytometry System (Becton Dickinson, San Jose, CA, USA).

### 4.7. TUNEL Assay

The terminal deoxynucleotidyl transferase dUTP nick-end labeling (TUNEL) assay was performed using a commercial fluorometric TUNEL system kit (KeyGEN BioTECH, Nanjing, China) according to the manufacturer’s instructions. MPC5 cells were seeded at a density of 2 × 10^4^ cells per well on coverslips in 24 wells. After transfection with SENP1 shRNA and negative control shRNA, cells were treated with 5 μg/mL PAN for 24 h. Nuclear DAPI staining and TUNEL staining were examined under fluorescence microscopy (Olympus, Center Valley, PA, USA) and assessed in ten random fields (200×) using Image Pro Plus software.

### 4.8. Measurement of Oxidative Stress

GSH/GSSG ratio is used to assess the exposure of cells to oxidative stress. We assessed the levels of GSH and GSSG of MPC5 cells and calculated the GSH/GSSG ratio using the total glutathione/oxidized glutathione assay kit (Jiancheng Bioengineering Institute, Nanjing, China), according to the protocols of the manufacturer.

### 4.9. Statistical Analysis

Values were expressed as the mean ± SEM. Statistical analysis was performed by ANOVA using a *post-hoc* analysis with Tukey’s test. The significance level was set at *p* < 0.05.

## 5. Conclusions

Our research revealed for the first time that SENP1-mediated deSUMOylation may be a potential target for the prevention of PAN-induced apoptosis in podocytes, at least in part by regulating the activity of the p53 pathway. This study provides a novel strategy for the treatment of podocyte apoptosis. However, the roles of SUMOylation and deSUMOylation in the pathogenesis of kidney diseases require further investigation.
